# Antiproliferative activity of antimicrobial peptides and bioactive compounds from the mangrove *Glutamicibacter mysorens*

**DOI:** 10.3389/fmicb.2023.1096826

**Published:** 2023-02-17

**Authors:** Yalpi Karthik, Manjula Ishwara Kalyani, Srinivasa Krishnappa, Ramakrishna Devappa, Chengeshpur Anjali Goud, Krishnaveni Ramakrishna, Muneeb Ahmad Wani, Mohamed Alkafafy, Maram Hussen Abduljabbar, Amal S. Alswat, Samy M. Sayed, Muntazir Mushtaq

**Affiliations:** ^1^Department of Studies and Research in Microbiology, Mangalore University, Mangalore, Karnataka, India; ^2^Department of Studies and Research in Biochemistry, Mangalore University, Mangalore, Karnataka, India; ^3^Dr. C.D Sagar Centre for Life Sciences, Biotechnology Department, Dayananda Sagar College of Engineering, Dayananda Sagar Institutions, Bengaluru, India; ^4^Department of Plant Biotechnology, School of Agricultural Sciences, Malla Reddy University, Hyderabad, India; ^5^Department of Studies and Research in Microbiology, Vijayanagara Sri Krishnadevaraya University, Ballari, Karnataka, India; ^6^Division of Floriculture, Sher-e-Kashmir University of Agricultural Sciences and Technology, Srinagar, Jammu and Kashmir, India; ^7^Department of Cytology and Histology, Faculty of Veterinary Medicine, University of Sadat City, Sadat City, Egypt; ^8^Department of Pharmacology and Toxicology, College of Pharmacy, Taif University, Taif, Saudi Arabia; ^9^Department of Biotechnology, College of Science, Taif University, Taif, Saudi Arabia; ^10^Department of Economic Entomology and Pesticides, Faculty of Agriculture, Cairo University, Giza, Egypt; ^11^ICAR-National Bureau of Plant Genetic Resources, Division of Germplasm Evaluation, New Delhi, India; ^12^MS Swaminathan School of Agriculture, Shoolini University of Biotechnology and Management, Bajhol, Himachal Pradesh, India

**Keywords:** anticancer, chromatography, FESEM, *Glutamicibacter mysorens*, mangrove soil, microbial peptides

## Abstract

The *Glutamicibacter* group of microbes is known for antibiotic and enzyme production. Antibiotics and enzymes produced by them are important in the control, protection, and treatment of chronic human diseases. In this study, the *Glutamicibacter mysorens* (*G. mysorens*) strain MW647910.1 was isolated from mangrove soil in the Mangalore region of India. After optimization of growth conditions for *G. mysorens* on starch casein agar media, the micromorphology of *G. mysorens* was found to be spirally coiled spore chain, each spore visualized as an elongated cylindrical hairy appearance with curved edges visualized through Field Emission Scanning Electron Microscopy (FESEM) analysis. The culture phenotype with filamentous mycelia, brown pigmentation, and ash–colored spore production was observed. The intracellular extract of *G. mysorens* characterized through GCMS analysis detected bioactive compounds reported for pharmacological applications. The majority of bioactive compounds identified in intracellular extract when compared to the NIST library revealed molecular weight ranging below 1kgmole^−1^. The Sephadex G-10 could result in 10.66 fold purification and eluted peak protein fraction showed significant anticancer activity on the prostate cancer cell line. Liquid Chromatography–Mass Spectrometry (LC–MS) analysis revealed Kinetin-9-ribose and Embinin with a molecular weight below 1 kDa. This study showed small molecular weight bioactive compounds produced from microbial origin possess dual roles, acting as antimicrobial peptides (AMPs) and anticancer peptides (ACPs). Hence, the bioactive compounds produced from microbial origin are a promising source of future therapeutics.

## Introduction

The environmental conditions in a particular ecosystem play an important role in determining biodiversity composition. High tides, hypersaline water, significant temperature fluctuations, and optimal flora and fauna diversity are just a few of the distinctive environmental characteristics of the mangrove ecosystem (Karthik et al., 2020). Microbes can better adapt to any extreme environment in these vulnerable situations. The isolation of bioactive chemicals will be greatly aided by this habitat (Alongi, 2015).

*Actinomyces* word derived from the words “atkis” which means “a ray” and “mykes” which means “fungi” are filamentous, Gram–positive bacteria distinguished by different coloration and spore production at maturity (Chater, 2006). *Actinomyces* share the characteristics of bacteria and fungi. The *Actinomyces* group’s genetic and environmental flexibility facilitates the development of worthwhile bioactive substances. *Actinomyces* contribute more in enzyme production to pharmacological industries for the treatment, and prevention of various ailments (Chater, 2013).

The pharmaceutical industry is constantly looking for drugs with innovative structures and new modes of action as a result of the rise in antibiotic resistance. There are still many environmental niches to investigate as potential sources of antibiotics (Karthik and Kalyani, 2021, 2022). One such *Actinomyces* group *Glutamicibacter* genus is broadly utilized in the control, treatment, and prevention of diseases through the production of bioactive compounds, widely used as antibiotics (Phuong and Diep, 2020), anti-tumor, anti-tubercular (Khusro et al., 2020), anti-helminthic, anti-diabetic, anti-oxidant from an exo-polysaccharide (Xiong et al., 2020; Fukuda and Kono, 2021; Hidri et al., 2022), anti-angiogenic, growth hormones (Qin et al., 2018; Hidri et al., 2022), immuno-suppressors, neuritogenic (Tang et al., 2021), anti-inflammatory (Hui et al., 2021), anti-algal (Agamennone et al., 2018), anti-fungal with enzymatic source (Mihooliya et al., 2017; Asif et al., 2020), anti-proliferative (Baig et al., 2021), anti-parasitic, anti-malarial, anti-viral, anti-bacterial and many more biological applications (Nishioka and Katayama, 1978; Renner et al., 1999; Fernebro, 2011; Janardhan et al., 2014; Desouky et al., 2015; Abd-Elnaby et al., 2016).

The various species of genus *Glutamicibacter* shown huge biological importance as detailed above. Whereas *G. creatinolyticus* shown resistance to antibiotics as well as heavy metals (copper, arsenic, cadmium, cobalt, zinc, and chromium; Santos et al., 2020). The *G. arilaitensis* produced pink colored pigment and coprophorphyrin binds zinc and regulates in cheese rinds (Cleary et al., 2018). Another *Gluamicibacter sps.* Possessing genes that regulates the growth of plant under saline conditions, cold adaptation, efficient degradation and chitinase enzyme producing genes which help in control the growth of pathogenic bacteria (Borker et al., 2021; Fu et al., 2021). While *G. nicotianae* involved in heavy metals degradation (Wang et al., 2021). The *G. mishrai* and *halophytocola* isolated from Andaman sea sample. Genes involved in cell wall biogenesis, replication, recombination, repair mechanism and amino acid metabolism along possess important role in physiology and behavior of insects (Qin et al., 2018; Das et al., 2020; Wang W, et al., 2022).

Antimicrobial peptides (AMPs) are peptides with antimicrobial properties. In multicellular organisms, these positively charged host defense molecules, or AMPs, serve as the initial line of protection. Many AMP’s from both prokaryotes and eukaryotes have been categorized (Brandenburg et al., 2012; Desriac et al., 2013). Several genera of AMP’s -producing microorganisms have been discovered, including bacteriocins produced by *Leuconostoc gelidum*, *Enterococcus faecium*, and other species (Juturu and Wu, 2018; Khodaei and Sh, 2018). Microcins A and B, antimicrobial bacteriocins derived from *Streptomyces pluripotens*, have been shown to be effective against *Escherichia coli, Salmonella typhimurium, Staphylococcus aureus,* and *Listeria monocytogenes* (Collin and Maxwell, 2019; Kurnianto et al., 2021)*.*These AMPs have been found to be effective in the treatment of a broad range of ailments (Sugrue et al., 2019; Karthik et al., 2020; Khadayat et al., 2020; Zhang et al., 2020). AMP’s are peptides derived from microbes that exhibit antimicrobial activity. AMPs have been shown to target cell walls or cell membranes, permitting them to penetrate cells and affect vital components while inhibiting growth (Desriac et al., 2013; Wang et al., 2020). As a result of their target-specific activity against resistant microbial species, AMPs are thought to be anti–microbial compounds.

Peptides with selective action and non-selective activity, i.e., those that have activity against bacteria, cancer cells, and healthy cells, can be categorized as having antitumor activity in Hoskin and Ramamoorthy’s investigations (Hoskin and Ramamoorthy, 2008).The peptides have antibacterial and anticancer properties, but not against normal cells. Cecropins, buforins, and magainins, among other peptides, have demonstrated anticancer effects without harming normal eukaryotic cells (Cruciani et al., 1991; Cho et al., 2009). These studies go into great detail and provide a compelling case for the fact that many peptides have biological activity in a variety of dimensions and properties and can possess dual activity as AMPs and ACPs. Therefore, we are searching for mangrove soil *Actinomyces* in the Mangalore region to isolate and characterize bioactive peptides that can function as both AMPs and ACPs.

In our previous study, we reported the detailed procedures for isolation, microscopic and macroscopic characters, identified as *Glutamicibacter mysorens* with GenBank accession number MW647910.1, the intracellular protein; extraction, estimation, along with their potential antimicrobial activity was observed against test pathogens *Salmonella typhimurium* (ATCC23564)*, Staphylococcus aureus* (ATCC6538P)*,Bacillus cereus* (ATCC10876)*, Proteus vulgaris* (ATCC13315)*,* and *Pseudomonas aeruginosa* (ATCC9027) cultures. The protein was characterized through LCMS and SDS PAGE techniques and small peptides were detected (Karthik and Kalyani, 2021).

In this study, the optimization of suitable growth media for *G. mysorens* and its micromorphology were analyzed using FESEM. The isolation of intracellular extract of *G. mysorens* was characterized through GCMS and LCMS. These GCMS studies revealed a large number of small bioactive compounds that possess significant biological activities are discussed. Whereas the LCMS studies resulted in the detection of low molecular weight Kinetin-9-ribose and Embinin showed significant anti-tumor potential against PC3 cell line in comparison to standard cisplatin drug.

## Materials and methods

### Mangrove soil collection

Soil samples were collected from Mangroves soil in Mangalore, Dakshina Kannada. Jeppinamogaru (JPMU) is located at 12°50′31.4”N 74°51′36.4″E. At the collecting site, the soil was brown with a powdery texture, and environmental parameters; the temperature of 21°C and pH of 7.2 was recorded. The collected samples were shifted to the Molecular Research Laboratory (MRL), Department of Microbiology, Jnana Kaveri, Mangalore University, India, in aseptic containers. To prevent fungal and bacterial growth, the soil sample was pre-heated for 2 h at 60°C prior to serial dilution and isolation (Mohan et al., 2013; Sridevi et al., 2015; Sapkota et al., 2020).

### Cultural characteristics

The isolated *G. mysorens* strain was subjected to FESEM analysis at different objectives distances; spore structure (1 and 2 μm) mycelial structure (10 and 20 μm) to visualize the complete micromorphological structures. The sequencing and identification of *G. mysorens* are reported by Karthik and Kalyani (2021).

### Intracellular extract

The *G. mysorens* strain was grown in SCN broth for 7 days at 30 ± 2°C with continuous shaking at 100 rpm. Centrifugation at 7000 rpm for 8 min separated the cultured biomass cells, which were then washed twice using phosphate-buffered saline devoid of Mg^2+^ and Ca^2+^ and centrifuged again. The cells were then re-suspended in 10 ml of chilled acetone for 5 min before being centrifuged at 7,000 rpm for 8 min. The intracellular extract was incubated for 2 min with 1.0 ml of 1% SDS after the traces of acetone was removed with a nitrogen stream (Bhaduri and Demchick, 1983). This intracellular extract was characterized using spectrometric (LCMS, GCMS) tools along with a comparison to the NIST library.

### Gas chromatography-mass spectroscopic analysis

The following equipment was assessed for the GC–MS studies of *G. mysorens* intracellular extract: a PerkinElmer Clarus 680 Gas Chromatograph and a PerkinElmer Clarus SQ 8C Mass Spectrometer. A PerkinElmer Elite-5MS standard column with dimensions of 30 m long x 0.250 mm inner diameter x 1 micron (60–350°C) is utilized in the equipment. With an equivalence ratio of 10:1, the injected volume of 2 μl was completely run for 26.6 min. Helium is used as the carrier gas, with a flow rate of 2 ml/min. The source temperature was set to 230 degrees Celsius, and the inlet temperature was set to 250 degrees Celsius. The oven temperature was initially set to 80°C with a hold time of 2.0 min; ramp1 was set to 10.0 /min to 150°C with a hold time of 1.0 min; and ramp2 was set to 15.0 /min to 250°C with a hold time of 10.0 min. The components were identified by comparing them to those contained in the NIST computer library, which was linked to the GC–MS apparatus, and the results were published.

### Gel filtration

The microbial proteins were purified using SephadexG-10. For 5 h, the Sephadex G-10 was allowed to swell in excess of dH_2_O in a boiling water bath. After decanting the gel to remove fines, it was equilibrated with 0.05 M sodium phosphate buffer, pH 7.0. Under gravity, the gel was packed into a 1.0 cm × 110.0 cm column. At a flow rate of 10 ml/h, the column was standardized with two-bed volumes of phosphate buffer of concentration 0.05 M, pH 7.0. The 20 mg of isolate protein sample was loaded onto the gel, eluted with 0.05 M sodium phosphate buffer, pH 7.0, and 2.0 ml fractions were collected and further analyzed (Bharadwaj et al., 2018).

### Liquid chromatography-mass spectrophotometer

The Sephadex G-10 peak fraction was analyzed using LC–MS, model Synapt G2, an analytical chemistry technique that combines the physical separation capabilities of liquid chromatography with mobile phases A: 0.1% Formic acid in Water and mobile phase B: 0.1% Formic acid in Acetonitrile with the mass analysis capabilities of mass spectrometry (MS) an Agilent 1100 LC system with a vacuum degasser, A BEH C18, 50 mm × 1.0 mm, 1.7 μm C18 column (Waters, United States) was used to achieve chromatographic separation in comparison to the NIST computer library.

### MTT assay

Prostate cancer cells (PC3) procured from NCCS Pune; were harvested in T-25 flasks for the *in vitro* studies. PC3 cells were trypsinized and aspirated into a 5 ml centrifuge tube. After centrifugation at 300 rpm for 10 min, the cell pellet was separated. The cell count was adjusted using DMEM HG medium so that 200 μl of suspension contained approximately 10,000 cells. In an ESCO model CLM170B-8-UV CO_2_ incubator, a 200 μl cell suspension was added to each well of the 96-well microtiter plate, and the plate was incubated for 24 h at 37°C and 5% CO_2_ atmosphere. After 24 h, the spent medium was aspirated. In each well, 200 μl of various test drug concentrations and the standard drug cisplatin were added. After that, the plates were incubated for 24 h at 37°C and 5% CO_2_. The drug-containing media was aspirated after the plate was removed from the incubator. The plate was then incubated for 3 h at 37\u00B0C and 5% CO_2_ atmosphere with 200 μl of medium containing 10% MTT reagent in each well to achieve a final concentration of 0.5 mg/ml. The culture medium was completely removed without disturbing the formed crystals. To solubilize the formed formazan, the plate was gently shaken in a gyrator shaker with 100 μl of solubilization solution (DMSO). The absorbance was read at 570 and 630 nm using the microplate reader of a Multiskan sky ELISA spectrophotometer.

## Results and discussion

The Mangrove region in Jeppinamogaru located at Mangalore, India, served as a suitable source for isolating *G. mysorens* strain. The *G. mysorens* strain received a GenBank accession number MW647910.1 and was isolated and their biological activities were reported by Karthik and Kalyani (2021). In continuation to previous work; initially, the *G. mysorens* strain was observed for morphological characteristics after performing FESEM analysis. Also, biologically important chemical components present in the intracellular extract of the *G. mysorens* strain were characterized using GCMS and a partially purified protein sample was characterized using LCMS and have a shown significant number of bioactive compounds.

**Figure 1:**
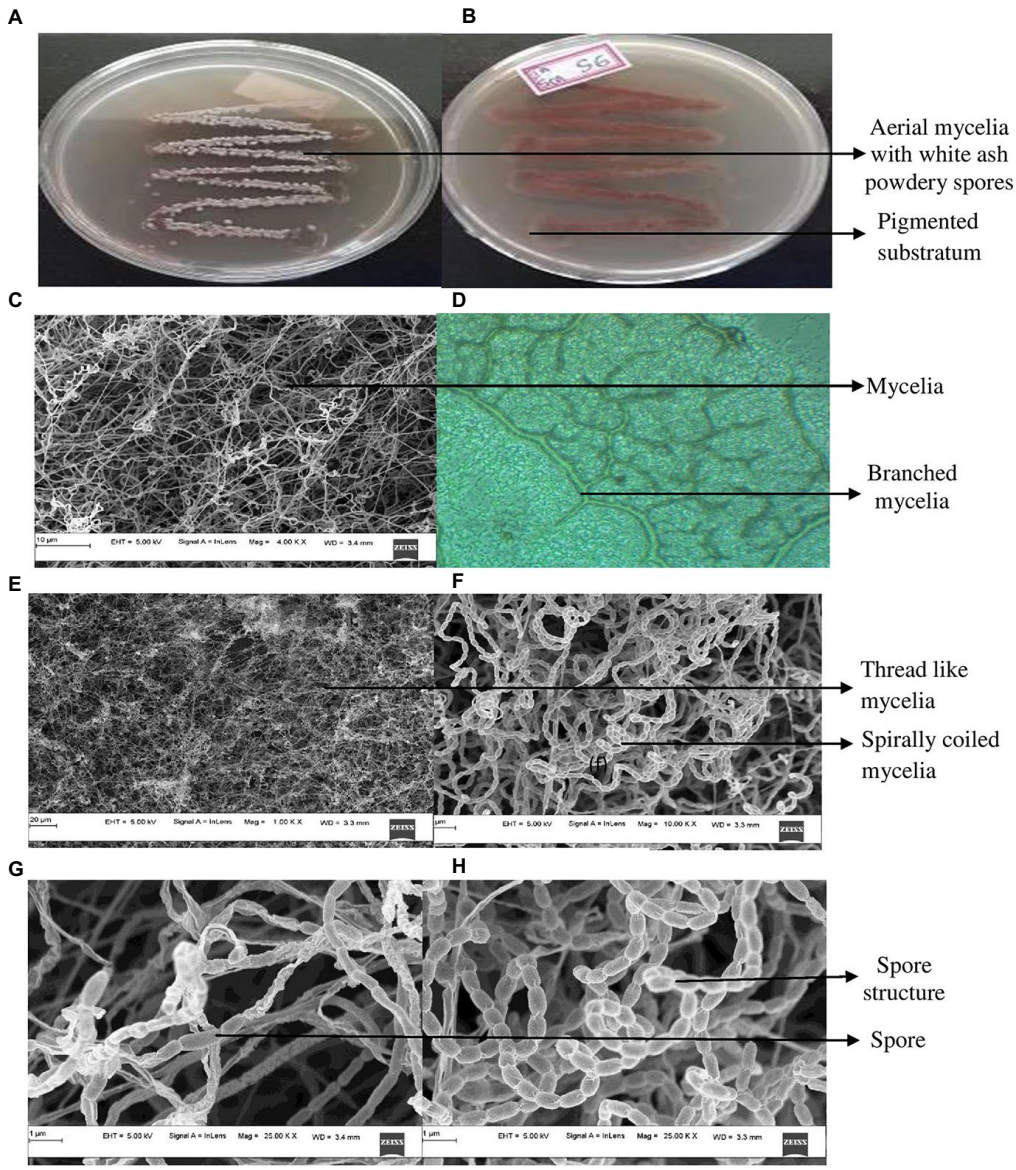
Cultural characteristics of *Glutamicibacter mysorens*. **(A)** Front view of isolate. **(B)** Rear view of isolate. **(C)** Mycelia observations under FESEM. **(D)** Phase contrast microscopic analysis. **(E,F)** Mycelia along with spore analysis under FESEM. **(G,H)** Spore structure analysis using FESEM.

The cultural characteristics of mangrove adapted *G. mysorens* strain upon growth on starch casein nitrate agar medium exhibited as white colored filamentous mycelia and at maturity showed ash-colored spores. Production of brown pigmentation on SCNA media was observed. Further microscopic analysis showed Gram staining positive. The isolate when further subjected to FESEM microscopic studies revealed mycelial morphological characteristics of the genus *Glutamicibacter.* Further, the culture showed filamentous mycelia possessing spirally coiled spore chains. Each spore is visualized as an elongated cylindrical hairy appearance with curved edges as shown in Figure 1. The *G. mysorens* when grown on different *Actinomyces*-specific media have shown distinctive phenotypic characteristics as listed in Table 1. Excellent growth was achieved on starch casein nitrate agar, whereas good growth was seen on, glucose leucine agar, yeast extract agar, and nutrient agar media. Moderate growth was seen on sucrose peptone agar, and malt extract agar.Whereas in another study, lysogeny agar was chosen as the best growth media for *G. mysorens* according to Wang Y. et al. (2022) and Deb et al. (2020).

**Table 1:** Phenotypic characteristics of *Glutamicibacter mysorens* on different media.

Media	Growth	Front view	Rear view	Pigment	Spores
Sucrose peptone agar	Moderate	Cream	Creamish white	−	No
Glucose luecine agar	Good	Cream	White	−	Black
Nutrient agar	Good	Creamish	Creamish	−	−
Malt extract agar	Moderate	Creamish white	Creamish white	−	No
Yeast extract agar	Good	Cream	Cream	−	White
Starch casein nitrate agar	Excellent	White ash	Brown	+	Grey

**Figure 2:**
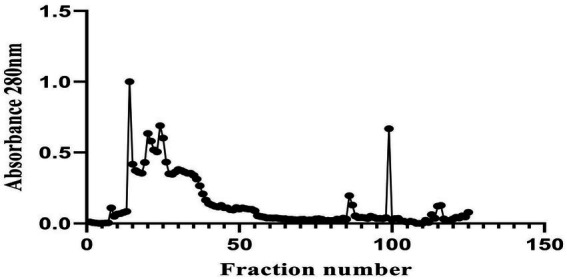
Elution profile of *Glutamicibacter mysorens* by using Sephadex G-10 column chromatography.

**Figure 3:**
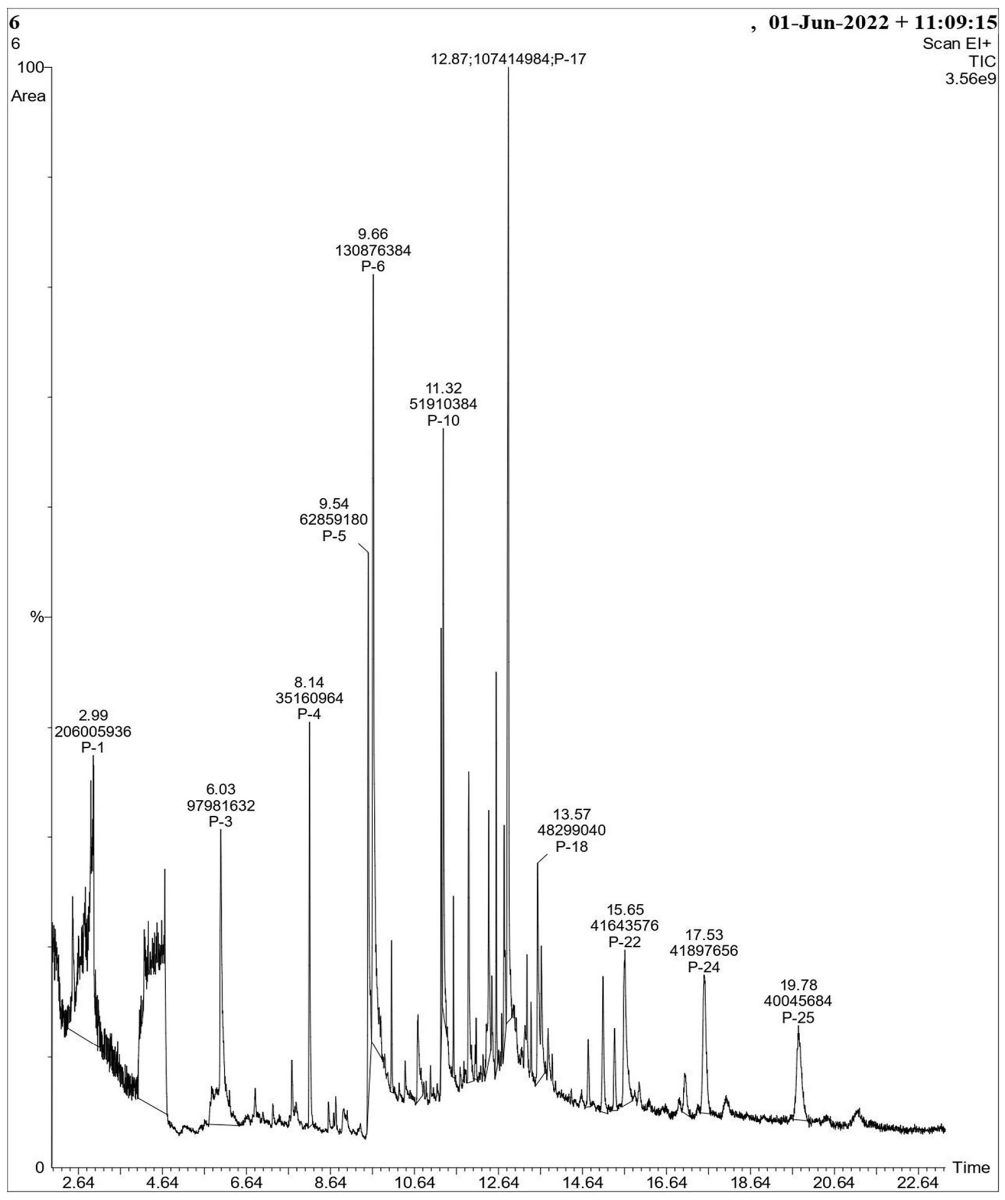
Elution profile of GCMS analysis for *Glutamicibacter mysorens* intracellular extract.

**Figure 4:**
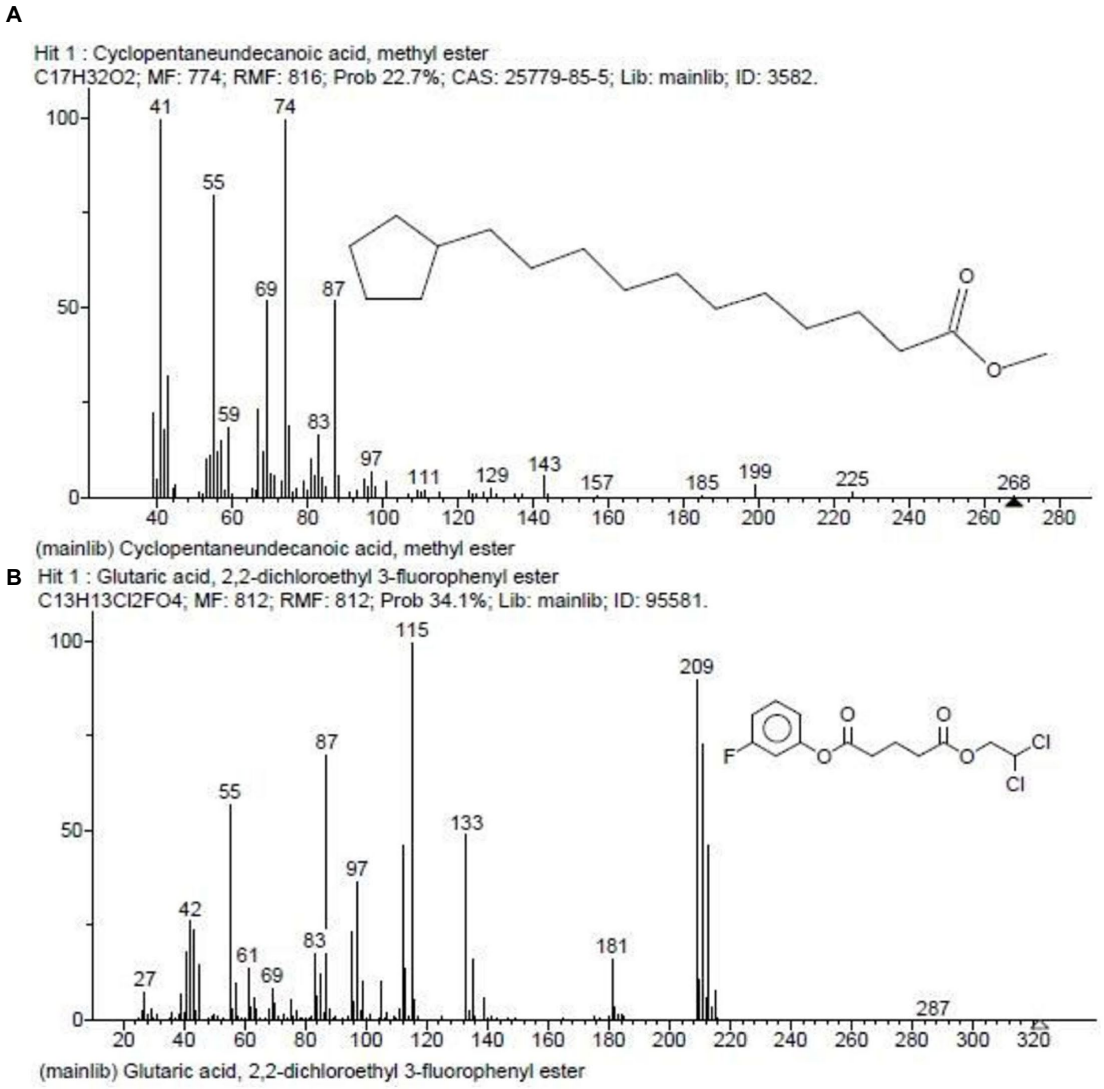
GCMS depicted highest probable compounds. **(A)** Cyclopentaneundecanoic acid, methyl ester 22.7%. **(B)** Glutaric acid, 2,2-dichloroethyl 3-fluorophenyl ester 34% probability.

In our previous report, the *G. mysorens* strain when subjected to simple and rapid disruption followed according to the method of Bhaduri yielded significant intracellular extraction in buffer (Bhaduri and Demchick, 1983). A 20 mg of protein was loaded on top of the column and 2 ml fractions were collected and about 2.5 times (216 ml) bed volumes of protein elutions were collected. The absorbance of protein fractions was checked at 280 nm and graphs were plotted. The X-axis indicates fraction numbers and absorbance plotted on Y-axis for each fraction collected from Sephadex G-10 column chromatography as showed in Figure 2. This column separation chromatography purifies 10.66 folds as detailed in Table 2. The GCMS studies depicted the presence of 155 bioactive molecules present in the intracellular extract of *G. mysorens* and the obtained elution profile is as shown in Figure 3. Whereas GCMS analysis depicted the highest probable compounds such as Cyclopentane undecanoic acid, methyl ester 22.7% and Glutaric acid, 2,2-dichloroethyl 3-fluorophenyl ester 34% probability as shown in Figure 4. All the compounds detected through GCMS showed low molecular weight below 1Kgmol^−1^with various pharmacological applications. The majority of bioactive compounds have shown antimicrobial, enzyme inhibitors, activators, antioxidants, anti-inflammatory, anticancer, agrochemical, insecticide, anti-obese, and many other applications as listed in Table 3. The intracellular extract of *G. mysorens* had shown potent antimicrobial activity to a broad spectrum of test pathogens such as *Salmonella typhimurium* (ATCC23564)*, Staphylococcus aureus* (ATCC6538P)*, Pseudomonas aeruginosa* (ATCC9027), *Proteus vulgaris* (ATCC13315)*,* and *Bacillus cereus* (ATCC10876) cultures. In order to focus further on prominent bioactive compounds the intracellular extract was partially purified using a Sephadex G-10 column. The eluted peak fraction upon spectrophotometry and electrophoretic analysis revealed the presence of peptide and is reported in our previous article (Karthik and Kalyani, 2021). A similar study was illustrated on 41 different *Actinomyces* species and majority isolates shown antagonist activity against *Staphylococcus aureus, Escherichia coli* and *Klebsiella pneumoniae* (Sapkota et al., 2020)*.*

**Table 2:** Purification chart of *Glutamicibacter mysorens* intracellular protein.

Sample	Protein (mg/ml)	Fold purification	% yield
Crude protein	2.0	1	100
Gel filtration (Sephadex G-10)	0.1875	10.66	9.38

**Table 3:** List of GC–MS analysis of bioactive compounds from *Glutamicibacter mysorens* intracellular extract.

Sl. No.	R.T (min)	Compound name	Activity/Applications	Molecular formula	Molecular weight (g/mol)	Area percentage	References
1	4.5	2-Pentanone, 4-hydroxy-4-methyl-	Photolysis	C_6_H_12_O_2_	116	0.9	Qiu et al. (2019)
2		Tert-Butyl Hydroperoxide	Oxidant	C_4_H_10_O_2_	90		Gad (2014)
3		1,3-Dioxolane-2-methanol, 2,4-dimethyl-	Chlorinating agent	C_6_H_12_O_3_	132		Simon and Losada (2008), Fuentes et al. (2016)
4		2-Propanol, 2-nitroso-, acetate	Cosmetics	C_5_H_9_NO_3_	131		Lemieux and Nagabhushan (1968)
5		2-Hexanone, 4-methyl-	Paints	C_7_H_14_O	114		Rebbert and Ausloos (1962)
6		2-Acetoxyisobutyryl chloride	Epoxides synthesis	C_6_H_9_ClO_3_	164		Zibuck (2001)
7	6.0	Octanoic acid, methyl ester	Oxidation	C_9_H_18_O_2_	158	8.6	Schwabe et al. (1964)
8		Undecanoic acid, 2-methyl-	Antifungal	C_12_H_24_O_2_	200		Rossi et al. (2021)
9		Methyl 6-methyl heptanoate	Biomolecule synthesis	C_9_H_18_O_2_	158		Kroumova and Wagner (2003)
10		Decanoic acid, methyl ester	Antibacterial	C_11_H_22_O_2_	186		Damiano et al. (2020)
11	6.8	Dodecanoic acid, 3-hydroxy-	Cytotoxic	C_12_H_24_O_3_	216	5.3	Viegas et al. (1989)
12		Oleic Acid	Anti-tumor	C_18_H_34_O_2_	282		Carrillo Perez et al. (2012)
13		12-Methyl-E,E-2,13-octadecadien-1-ol	Antioxidant	C_19_H_36_O	280		Salem et al. (2016)
14		Z-8-Methyl-9-tetradecenoic acid	Antibacterial	C_15_H_28_O_2_	240		Jawad et al. (2016)
15		Z-(13,14-Epoxy)tetradec-11-en-1-ol acetate	Anti-inflammatory	C_16_H_28_O_3_	268		Abdul et al. (2020)
16		trans-13-Octadecenoic acid/ cis-Vaccenic acid	Anti-protozoal/ Protects from Heart failure	C_18_H_34_O_2_	282		Carballeira et al. (2009), Djoussé et al. (2014)
17		7-Hexadecenoic acid, methyl ester, (Z)-	Antioxidant	C_17_H_32_O_2_	268		Reza et al. (2021)
18		1-Octanol, 2,7-dimethyl-	Antioxidant, hepatoprotective and anti-inflammatory	C_10_H_22_O	158		Bentley et al. (2002)
19		Carbonic acid, prop-1-en-2-yl undecyl ester	Beverages production	C_15_H_28_O_3_	256		Millero et al. (2006)
20		1-Decanol, 2-ethyl-	Surfactant	C_12_H_26_O	186		Achimon et al., 2022
21		1-Decanol, 2-methyl-	Lubricants, Plasticizers	C_11_H_24_O	172		Halling et al. (1998)
22		Trichloroacetic acid, decyl ester	Disinfectant	C_12_H_21_Cl_3_O_2_	302		Anand et al. (2014)
23		1-Heptanol, 2-propyl-	Pheromone	C_10_H_22_O	158		Francke and Schulz (1999)
24		1-Octanol, 2-butyl-	Antioxidant	C_12_H_26_O	186		Abdillah et al. (2015)
25		Carbonic acid, decyl prop-1-en-2-yl ester	Beverages production	C_14_H_26_O_3_	242		Millero et al. (2006)
26	7.2	1,7-Octanediol, 3,7-dimethyl-	Polymer	C_10_H_22_O_2_	174	8.6	Reddy and Ananthaprasad (2021)
27		Octanoic acid, 7-oxo−/ Methyl 6-oxoheptanoate	Antibacterial	C_8_H_14_O_3_	158		Schwabe et al. (1964)
28		1,8-Nonanediol, 8-methyl-	Agrochemicals	C_10_H_22_O2	174		Kula et al. (2001)
29		7-Octen-2-ol, 2,6-dimethyl-	Cosmetics	C_10_H_20_O	156		Ham and Raymond Wells (2009)
30		3-Heptanol, 4-methyl-	Therapeutics	C_8_H_18_O	130		Ley and Madin (1991)
31		4-Heptanone, 2,3:5,6-diepoxy-2,6-dimethyl-	Oxidant	C_9_H_14_O_3_	170		Ley and Madin (1991)
32		3-Tridecanol	Lubricant	C_13_H_28_O	200		Chagnes et al. (2010)
33		2-Dodecanone	Insecticide	C_12_H_24_O	184		Wang et al. (2019)
34	7.7	3-(Prop-2-enoyloxy)dodecane	Antibiotics	C15H28O2	240	5.3	Fadhil et al. (2018)
35		3-(Prop-2-enoyloxy)tetradecane	Phyto-constituent	C_17_H_32_O_2_	268		Ezekwe et al. (2020)
36		2-Propenoic acid, 1-methylundecyl ester	Antibacterial	C15H28O2	240		Deryabin and Tolmacheva (2015)
37		5-(Prop-2-enoyloxy)pentadecane	Antimicrobial	C_18_H_34_O_2_	282		Xue et al. (2017), Gadhi et al. (2019)
38		3-Cyclopropylcarbonyloxydodecane	Reducing Agent	C_16_H_30_O_2_	254		Bolade et al. (2018)
39		9-Methyl-Z-10-pentadecen-1-ol	Antioxidant	C_16_H_32_O	240		Soleha et al. (2020)
40		Octadecane, 1-(ethenyloxy)-	Anti-corrosion	C_20_H_40_O	296		Zeitoun et al. (2021)
41		Dodecyl acrylate	Polyemerization	C_15_H_28_O_2_	240		Buback and Kowollik (1999)
42		Octanoic acid, 2-propenyl ester	Antioxidant	C_11_H_20_O_2_	184		Windey et al. (2012)
43	8.7	Octadecane, 6-methyl-	Enzymatic	C_19_H_40_	268	4.1	Holman et al. (1966)
44		Hydroxylamine, O-decyl-	Reducing agent	C_10_H_23_NO	173		(Gad, 2014)
45		Tetradecane, 2,6,10-trimethyl-	Hydrocarbon	C_17_H_36_	240		McCarthy and Calvin (1967)
46		Silane, trichlorodocosyl-	Surfactant	C_22_H_45_Cl_3_Si	442		Janneck et al. (2018)
47		Nonadecane	Binding material	C_19_H_40_	268		Li et al. (2010)
48		Oxirane, [(hexadecyloxy)methyl]-	Antibacterial	C_19_H_38_O_2_	298		Es (2014)
49		Decane, 1,1′-oxybis-	Antimicrobial	C_20_H_42_O	298		Fauzi et al. (2017)
50		1-Hexadecanol, 2-methyl-	Antioxidant	C_17_H_36_O	256		Hussein et al. (2015)
51		4-Hydroxy-4-methylhex-5-enoic acid, tert.-butyl ester	Hydrocarbon	C_11_H_20_O_3_	200		Ming Miao and Zhi (2018)
52		Z,Z-2,5-Pentadecadien-1-ol	Pharmacological	C_15_H_28_O	224		Millero et al. (2006)
53		l-Gala-l-ido-octose	Neuritogenic, Anti-hyper cholesteromia	C_8_H_16_O_8_	240		Jahan et al. (2020)
54		2-Cyclopropylcarbonyloxytridecane	aphrodisiac, anti-inflammatory, antihypertensive	C_17_H_32_O_2_	268		Sridhar et al. (2016)
55		Imidazole, 2-amino-5-[(2-carboxy)vinyl]-	Therapeutic	C_6_H_7_N_3_O_2_	153		Shalini et al. (2010)
56	9.5	4-Ethylacridine/3H-indole, 2-methyl-3-phenyl-	Antioxidant	C_15_H_13_N	207	4.2	Hosseini Hashemi et al. (2015)), Britten and Smith (1972)
57							
58		4-Pyridinol 3,5-dichloro-2-ethyl-6-methyl-	Herbicide	C_8_H_9_Cl_2_NO	205		Ransom et al. (2012)
59		5-Methyl-2-phenylindolizine/3-Methyl-2-phenylindole/2-Methyl-7-phenylindole	Antimicrobial, Antioxidant	C_15_H_13_N	207		Onocha et al. (2011)
60		Pyridine, 2,4-dichloro-5-thiocyanato-	Antimicrobial	C_6_H_2_Cl_2_N_2_S	204		Al-Salahi et al. (2010)
61		Dichloroacetic acid, phenyl ester/ Benzoic acid, 2,5-dichloro-, methyl ester	Therapeutic	C_8_H_6_Cl_2_O_2_	204		Babar et al. (2008)
62		3,5-Dichloro-2,4-dimethyl-1-methoxybenzene	Anticancer	C_9_H_10_Cl_2_O	204		Dhakal et al. (2020)
63		1-Chloroundecane	Precursor for fatty acid synthesis	C_11_H_23_Cl	190		Gensler and Thomas (1952)
64		Dodecane, 1-chloro-	Hydrocarbon	C_12_H_25_Cl	204		Moldoveanu (2019)
65		Tetradecane, 1-chloro-	Chlorination	C_14_H_29_Cl	232		Assassi et al. (2005)
66		Nonane, 1-chloro-	Hydrocarbon	C_9_H_19_Cl	162		Moldoveanu (2019)
67	10.0	Benzene, 1,4-bis(trifluoromethyl)-	Flurochrome	C_8_H_4_F_6_	214		Skhirtladze et al. (2022)
68		Pyrimidine, 4,5-diamino-6-chloro-2-(trifluoromethyl)-	Transcriptional activator	C_5_H_4_ClF_3_N_4_	212		Palanki et al. (2000)
69		1H-Imidazole, 1-(2,2,3,3,3-pentafluoro-1-oxopropyl)-	Anticancer	C_6_H_3_F_5_N_2_O	214		Zhang et al. (2014)
70		Sulfaguanidine	Enzyme inhibitor	C_7_H_10_N_4_O_2_S	214		Akocak et al. (2021)
71		Anthracene, 2-chloro-	Antibacterial	C_14_H_9_Cl	212		de Bony et al. (1984)
72		Ethyl iodoacetate	Enzyme activator	C_4_H_7_IO_2_	214		Tanaka and Hayashi (2008)
73		8-Methyl-4-(1-pyrrolidinyl)pyrido[3,2-c]pyridazine	Cancer therapies	C_12_H_14_N_4_	214		Jubete et al. (2019)
74		[1,1’-Biphenyl]-4-carboxylic acid, 4′-hydroxy-	Precursor for synthesis of bioactive molecules	C_13_H_10_O_3_	214		Patel et al. (2004)
75		Benzoic acid, 2-(1,2,4-triazol-3-yl-aminocarbonyl)-	Breast and prostate cancer therapy	C_10_H_8_N_4_O_3_	232		Jamieson et al. (2012)
76		Succinic acid, 2-methylpent-3-yl pentafluorobenzyl ester	Antioxidant	C_17_H_19_F_5_O_4_	382		Cullere et al. (2004)
77		1,1’-Biphenyl, 2-iodo-	Substrate	C_12_H_9_I	280		Fang et al. (2017)
78		Benzamide, N-(1,4,6-trimethyl-1H-pyrazolo[3,4-b]pyridin-3-yl)-	Substrate	C_16_H_16_N_4_O	280		Jachak et al. (2006)
79		4-[N′-(4-Methoxy-benzoyl)-hydrazino]-4-oxo-butyric acid methyl ester	Antibacterial	C_13_H_16_N_2_O_5_	280		EL-Hashash et al. (2014)
80		Dibenzo[a,c]phenazine	Flurochrome	C_20_H_12_N_2_	280		Xie et al. (2019)
81		Benzofuro[3,2-d]pyrimidine, 4-(2-pyridylthio)-	Therapeutic	C_15_H_9_N_3_OS	279		Campos et al. (2022)
82		(9E)-Styrylanthracene	Luminophore	C_22_H_16_	280		Zhang et al. (2017)
83		1H-Purine-2,6-dione,3,7-dihydro-3-methyl-7-carboxymethyl-8-n-butyl	Anti-inflammatory	C_12_H_16_N_4_O_4_	280		Abou-Ghadir et al. (2014)
84		Methyl 2-phenyl-2,3-epoxyindan-1-one-3-carboxylate	Catalyst	C_17_H_12_O_4_	280		Godwin et al. (2012)
85		Propyl N-(heptafluorobutyryl)pyroglutamate	Metabolite	C_12_H_12_F_7_NO_4_	367		Hušek et al. (2016)
86	10.4	3-Trifluoroacetoxypentadecane	Antimicrobial	C_17_H_31_F_3_O_2_	324	1.3	Hussein et al. (2015)
87		3-Cyclopropylcarbonyloxytetradecane	Antioxidant, Cytotoxic and Antibacterial	C_18_H_34_O_2_	282		Upgade and Bhaskar (2013)
88		10-Undecenoic acid, octyl ester	Antimicrobial	C_19_H_36_O_2_	296		Van der Steen and Stevens (2009)
89		3-(Prop-2-enoyloxy)tetradecane	Antioxidant	C17H32O2	268		Ezekwe et al. (2020)
90		Z-10-Tetradecen-1-ol acetate	Pharmaceutical	C_16_H_30_O_2_	254		Bolade et al. (2018)
91		5-Amino-2-methoxy-4-(1H-1,2,3,4-tetrazol-5-yl)phenol	Antimicrobial	C_8_H_9_N_5_O_2_	207		Arulmurugan and Kavitha (2010)
92		4H-Pyrido[1,2-a]pyrimidine-3-carboxamide, 6,7,8,9-tetrahydro-6-methyl-4-oxo-	Antimicrobial and antitumor	C_10_H_13_N_3_O_2_	207		Al-Taisan et al. (2010)
93		1-Adamantanecarboxamide, N,N-dimethyl−/ Pent-3-yn-2-ol, 2-cyclopropyl-5-(1-piperidyl)	Anticancer	C_13_H_21_NO	207		Su et al. (2012)
94		trans-4-Ethoxy-β-methyl-β-nitrostyrene/ Carbamic acid, 4-methoxyphenyl-, allyl ester	Cardiovascular therapy	C_11_H_13_NO_3_	207		Alves-Santos et al. (2019)
95		Thiophen-2-methylamine, N-(2-fluorophenyl)-	Catalytic activity	C_11_H_10_FNS	207		Tanak et al. (2020)
96		2-(1-Piperidino)-3-nitropyridine	Antimicrobial	C_10_H_13_N_3_O_2_	207		Sivaprakash et al. (2019)
97		Benzoic acid, 4-amino-, pentyl ester	Cytotoxicity	C_12_H_17_NO_2_	207		Kratky et al. (2019)
98	10.5	Cyclopentaneundecanoic acid, methyl ester	Antioxidant and Antibacterial	C_17_H_32_O_2_	268	1.3	Daniels and Temikotan (2021)
99		Undecanoic acid, 10-methyl-, methyl ester	Antioxidant	C_13_H_26_O_2_	214		Narra et al. (2017)
100		Methyl 8-methyl-nonanoate	Antimicrobial and Anti-inflammatory	C_11_H_22_O_2_	186		Kaur et al. (2022)
101		Tetradecanoic acid, 12-methyl-, methyl ester	Larvicidial	C_16_H_32_O_2_	256		Xu et al. (2008)
102		Cyclopentanetridecanoic acid, methyl ester	Cytotoxic	C_19_H_36_O_2_	296		Joshi et al. (2020)
103	10.7	Glutaric acid, 2,2-dichloroethyl 3-fluorophenyl ester	Anti-angiogenic	C_13_H_13_Cl_2_FO_4_	322	1.0	Amaral et al. (2021)
104		Triethylgermanium bromide	Oxidant	C_6_H_15_BrGe	240		Satgé et al. (1973)
105		2,5-Cyclohexadien-1-one, 2,6-dichloro-4-(chloroimino)−/ benzene, 1,3,5-trichloro-2-nitroso-	Surfactant	C_6_H_2_Cl_3_NO	209		Yamamoto (2002)
106		Pyridine, 3,4,5-trichloro-2,6-dimethyl-	Antimicrobial	C_7_H_6_Cl_3_N	209		Khidre et al. (2011)
107		Ethaneselenoamide, N-(4-methylphenyl)-	Anticancer	C_9_H_11_NSe	213		Watanabe et al. (1997)
108		Stannane, chlorotriethyl-	Polymerization	C_6_H_15_ClSn	242		Qiu et al. (2013)
109		1-(2,4,5-Trichlorophenyl)ethanol	Cytotoxic	C_8_H_7_Cl_3_O	224		Shawky et al. (2021)
110		1,3-Dioxolane, 2-(5,5,5-trichloro-3-penten-1-yl)-, (E)-	Flavoring agent	C_8_H_11_Cl_3_O_2_	244		Ivankin (2017)
111		benzene, 1,1′-[oxybis(methyleneoxy)]bis[2,4,6-trichloro-	Toxic agent	C_14_H_8_Cl_6_O_3_	434		Holman et al. (1966)
112	11.5	Undecanoic acid	Antifungal	C_11_H_22_O_2_	186	0.9	Rossi et al. (2021)
113		n-Decanoic acid	Beverage production	C_10_H_20_O_2_	172		Viegas et al. (1989)
114		n-Hexadecanoic acid	Anti-inflammatory	C_16_H_32_O_2_	256		Aparna et al. (2012)
115		4-(Benzoylmethyl)-6-methyl-2H-1,4-benzoxazin-3-one	Antimicrobial	C_17_H_15_NO_3_	281		Ozden et al. (2000)
116		Adenine, N4-pentafluoropropionyl-	Oxidization	C_8_H_4_F_5_N_5_O	281		Tsunoda et al. (2011)
117		2-Furancarboxylic acid, N′-[(8-hydroxy-5-quinolinyl)methylidene]hydrazide	Antioxidant	C_15_H_11_N_3_O_3_	281		Gülerman et al. (2000)
118		1-Phenyl-4-(trifluoromethyl)-1H,4H,5H,6H,7H-pyrazolo[3,4-b]pyridin-6-one	Antiproliferative	C_13_H_10_F_3_N_3_O	281		Martín-Acosta et al. (2021)
119		Acetamide, 2-(2,4-difluorophenoxy)-N-(4-fluorophenyl)-	Inhibitor	C_14_H_10_F_3_NO_2_	281		Williams et al. (2015)
120		Succinic acid, 3,5-dinitrobenzyl 2-methylhex-3-yl ester	Enzyme activator	C_18_H_24_N_2_O_8_	396		Martinez et al. (2008)
121		Oxalic acid, monoamide, N-(2-fluorophenyl)-, heptyl ester	Antioxidant	C_15_H_20_FNO_3_	281		Ganyam et al. (2019)
122		Propanamide, 2,2,3,3,3-pentafluoro-N-(2,4,6-trimethylphenyl)-	Inhibitor	C_12_H_12_F_5_NO	281		Talley et al. (2000)
123	12.3	3-Trifluoroacetoxydodecane	Antioxidant	C_14_H_25_F_3_O_2_	282		Zagulyaeva et al. (2010)
124	12.5	Cyclopropanepentanoic acid, 2-undecyl-, methyl ester, trans-	Anti-mycobacterial	C_20_H_38_O_2_	310	1.5	Carballeira et al. (2007)
125		13,16-Octadecadiynoic acid, methyl ester	Antioxidant	C_19_H_30_O_2_	290		Hamalainen et al. (2001)
126		13-Tetradecynoic acid, methyl ester	Anti-inflammatory	C_15_H_26_O_2_	238		James and Martin (1956)
127		Oxiraneundecanoic acid, 3-pentyl-, methyl ester, cis-	Antimicrobial	C_19_H_36_O_3_	312		Al-Marzoqi et al. (2016)
128		9-Octadecenoic acid (Z)-, methyl ester/11-Octadecenoic acid, methyl ester	Food and Pharmacological	C_19_H_36_O_2_	296		Jiang and Jia (2015)
129		13-Docosenoic acid, methyl ester	Food industires	C_23_H_44_O_2_	352		Beare-Rogers (1977)
130	13.2	Z-(13,14-Epoxy)tetradec-11-en-1-ol acetate	Anti-inflammatory	C_16_H_28_O_3_	268	2.0	Abdul et al. (2020)
131		12-Methyl-E,E-2,13-octadecadien-1-ol/2-Methyl-Z,Z-3,13-octadecadienol	Therapeutic	C_19_H_36_O	280		Adeyemi (2017)
132		Z-8-Methyl-9-tetradecenoic acid	Antimicrobial	C_15_H_28_O_2_	240		Jawad et al. (2016)
133		Oxiraneoctanoic acid, 3-octyl-, cis-	Antimicrobial	C_18_H_34_O_3_	298		Hussein et al., 2016
134		Pentadecanoic acid	Oxidation	C_15_H_30_O_2_	242		Jenkins et al. (2015)
135		Heptadecanoic acid, heptadecyl ester	Antimicrobial	C_34_H_68_O_2_	508		Gautam et al. (2016)
136		2-Myristynoyl pantetheine	Antimicrobial	C_25_H_44_N_2_O_5_S	484		Srivastava et al. (2015)
137		9-Octadecenoic acid, (E)-	Inhibitor	C_18_H_34_O_2_	282		Carrillo Perez et al. (2012)
138		9-Hexadecenoic acid/1,2-15,16-Diepoxyhexadecane	Cosmetics	C_16_H_30_O_2_	254		Takigawa et al. (2005)
139		cis-13-Eicosenoic acid	Anti-obesity	C_20_H_38_O_2_	310		Senarath et al. (2018)
140		3-Heptafluorobutyroxytetradecane	Polymerization	C_18_H_29_F_7_O_2_	410		MacKenzie and Tenaschuk (1979)
141		n-Nonadecanol-1	Antifeedant	C_19_H_40_O	284		Aznar-Fernandez et al. (2019)
142	14.7	Hexanedioic acid, mono(2-ethylhexyl)ester	Antibacterial	C_14_H_26_O_4_	258	0.4	Choi and Jiang (2014)
143		Hexanedioic acid, dioctyl ester	Inhibitor	C_22_H_42_O_4_	370		Chaler et al. (2004)
144		Cyclohexanecarboxylic acid, octyl ester		C_15_H_28_O_2_	240		Andersson et al. (1965)
145		1-Dodecanol, 3,7,11-trimethyl-	Cytotoxic	C_15_H_32_O	228		Fahem et al. (2020)
146		Cyclohexanecarboxylic acid, decyl ester/2-Propenoic acid, tetradecyl ester	Antioxidant	C_17_H_32_O_2_	268		Matthew et al. (2022)
147		Hexanedioic acid, bis(2-ethylhexyl) ester	Biomarker	C_22_H_42_O_4_	370		Silva et al. (2013)
148	15.3	10-Octadecenal/4-Octadecenal	Adjuvant/ pheromones	C_18_H_34_O	266	0.4	Gil et al. (1995)
149		Cyclopropanetetradecanoic acid, 2-octyl-, methyl ester	Pharmacological	C_26_H_50_O_2_	394		Srivastava et al. (2015)
150		9-Methyl-Z-10-pentadecen-1-ol	Antioxidant	C_16_H_32_O	240		Soleha et al. (2020)
151		Hexadecane, 1,1-bis(dodecyloxy)-		C_40_H_82_O_2_	594		Ser et al. (2015)
152		3-Chloropropionic acid, heptadecyl ester	Antibiotic	C_20_H_39_ClO_2_	346		Ikhsanov et al. (2018)
153		2-Tridecenoic acid, (E)-	Antimicrobial	C_13_H_24_O_2_	212		Chowdhury et al. (2021)
154		trans-2-undecenoic acid	Larvicidal	C_11_H_20_O_2_	184		Saxena and Stotzky (2001)
155		Ethanol, 2-(octadecyloxy)-	Antimicrobial	C_20_H_42_O_2_	314		Jaffar et al. (2015)

* R.T (min): Retention Time.

**Figure 5:**
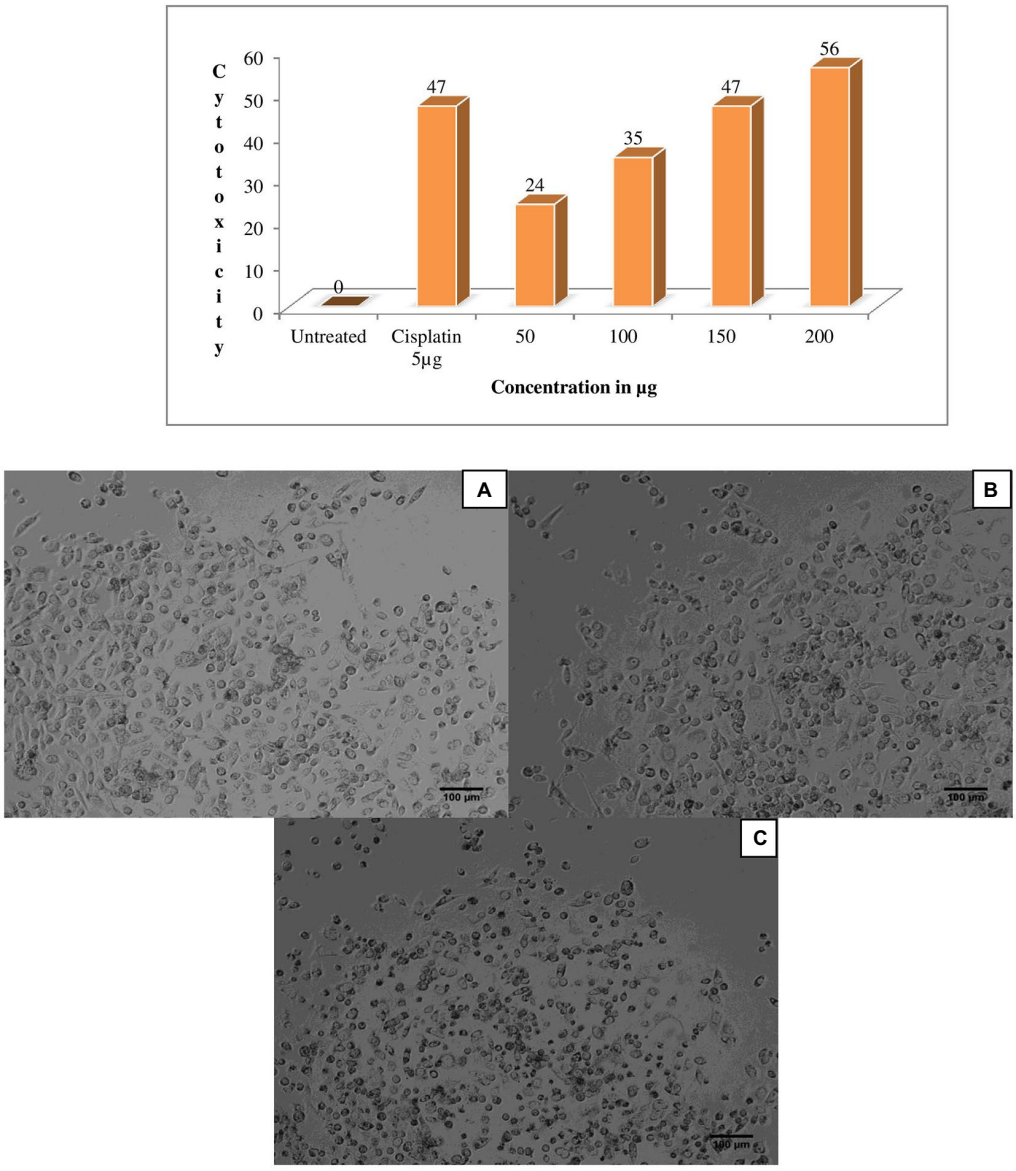
Anticancer activity of *Glutamicibacter mysorens* strain protein. MTT assay performed by using prostate cancer PC3 cell line. **(A)** Untreated cells of PC3 cell line, **(B)** Standard cisplatin at 5 μg/ml, **(C)** 56% Anticancer activity of *Glutamicibacter mysorens* protein at 200 μg/ml.

One of the previous study; extracellular protein of *Actinomyces* are actively producers for enzyme ligno cellulase (Clark Mason et al., 1988). The eluted peak fraction for proteins of *G. mysorens* has shown significant activity for different concentrations 50 μg of protein fraction showed 24% antiproliferative activity against prostate cancer PC3 cell line, for 100 μg 35% antiproliferative activity was observed, for 150 μg 47% antiproliferative activity was observed and for 200 μg 56% antiproliferative activity was observed in comparison with standard drug cisplatin at 5 μg showed 47% antiproliferative activity as showed in Figure 5.

Similar studies reported that other bioactive compounds isolated from the genus *Glutamicibacter* have been characterized for antimicrobial activity (Phuong and Diep, 2020; Xiong et al., 2020). In another study reported that plant-growth promoting bioactive compounds was produced by *Glutamicibacter halophytocola* coastal region of China (Qin et al., 2018). Whereas another study describes the anti-fungal efficiency of the *Glutamicibacter* genus with chitin hydrolyzing activity (Asif et al., 2020). The intracellular protein extraction already reported in our previous studies characterized for an antimicrobial activity that can be considered as antimicrobial peptides (AMPs) from the microbial origin (Karthik and Kalyani, 2021). In the present work the *G. mysorens* protein fraction is also exhibiting antiproliferative activity against cancerous cells acting also as anticancer peptides (ACP’s) and the protein molecules detected and characterized by LC–MS analysis. We are also reporting GCMS analysis and detected bioactive compounds from *G. mysorens*.

**Figure 6:**
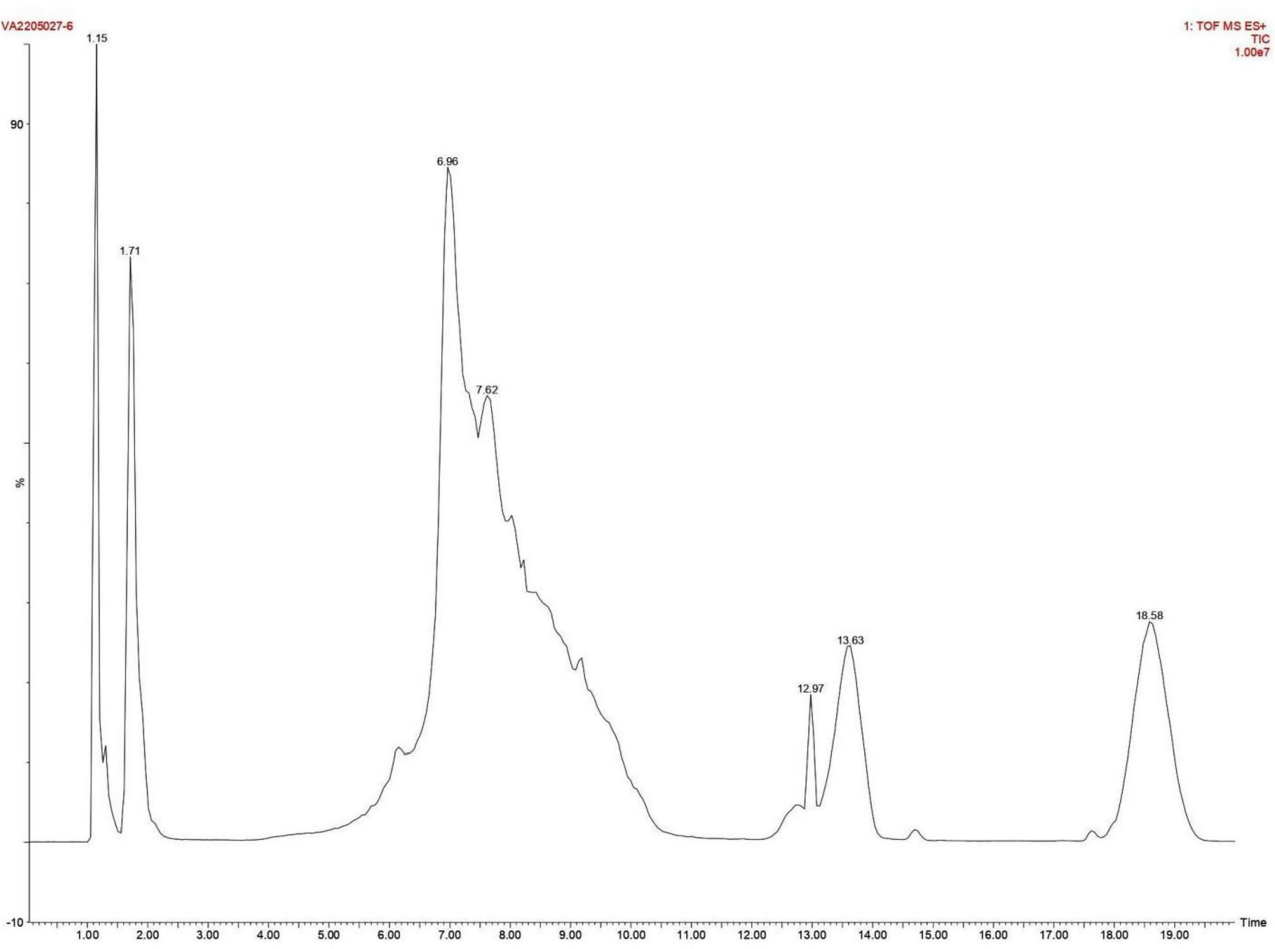
Elution profile of intracellular protein extract from *Glutamicibacter mysorens* by LCMS.

**Figure 7:**
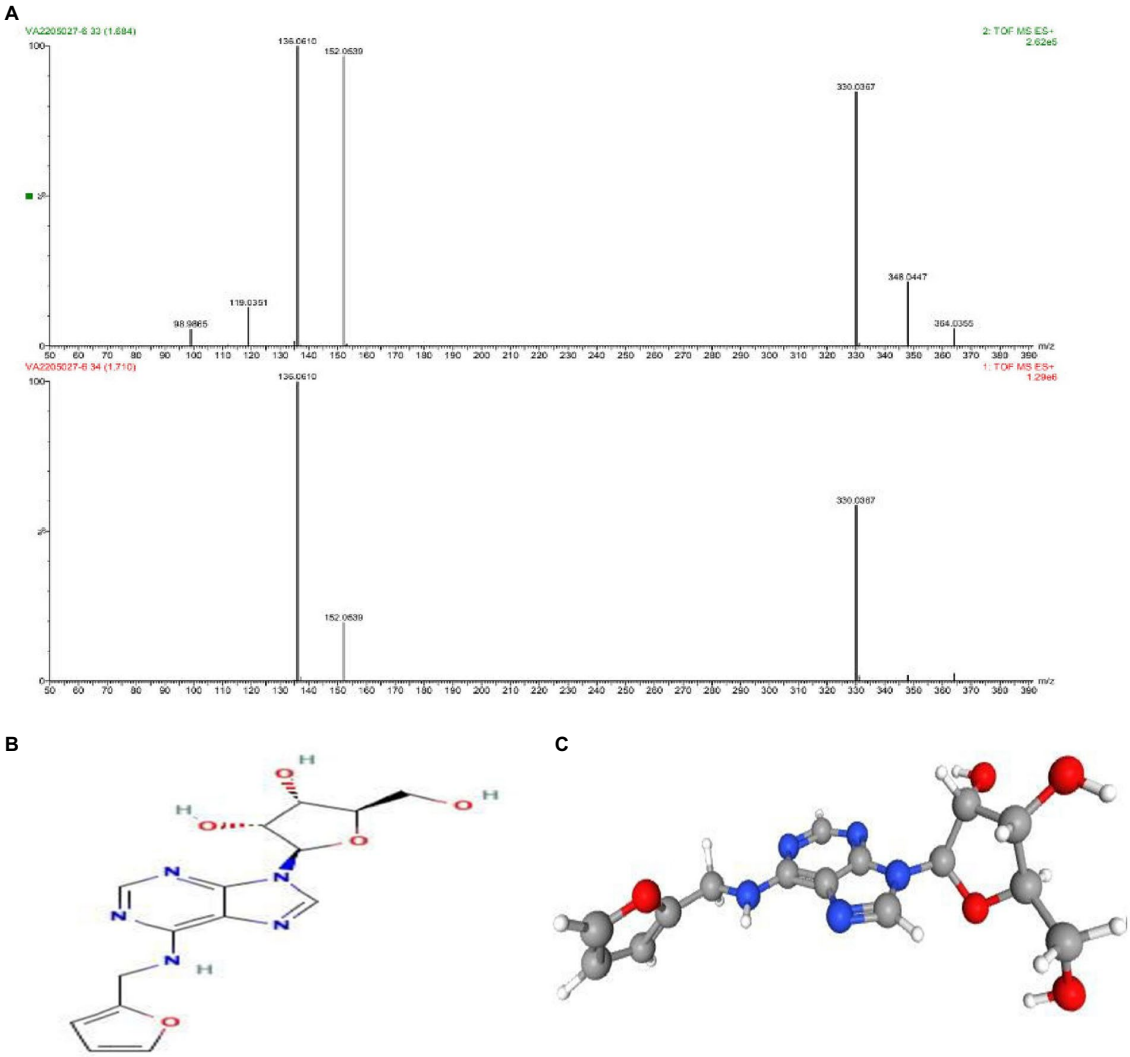
**(A)** Mass confirmation and analysis record. **(B)** 2D structure of kinetin-9-ribose, **(C)** 3D structure of kinetin-9-ribose molecule.

**Figure 8:**
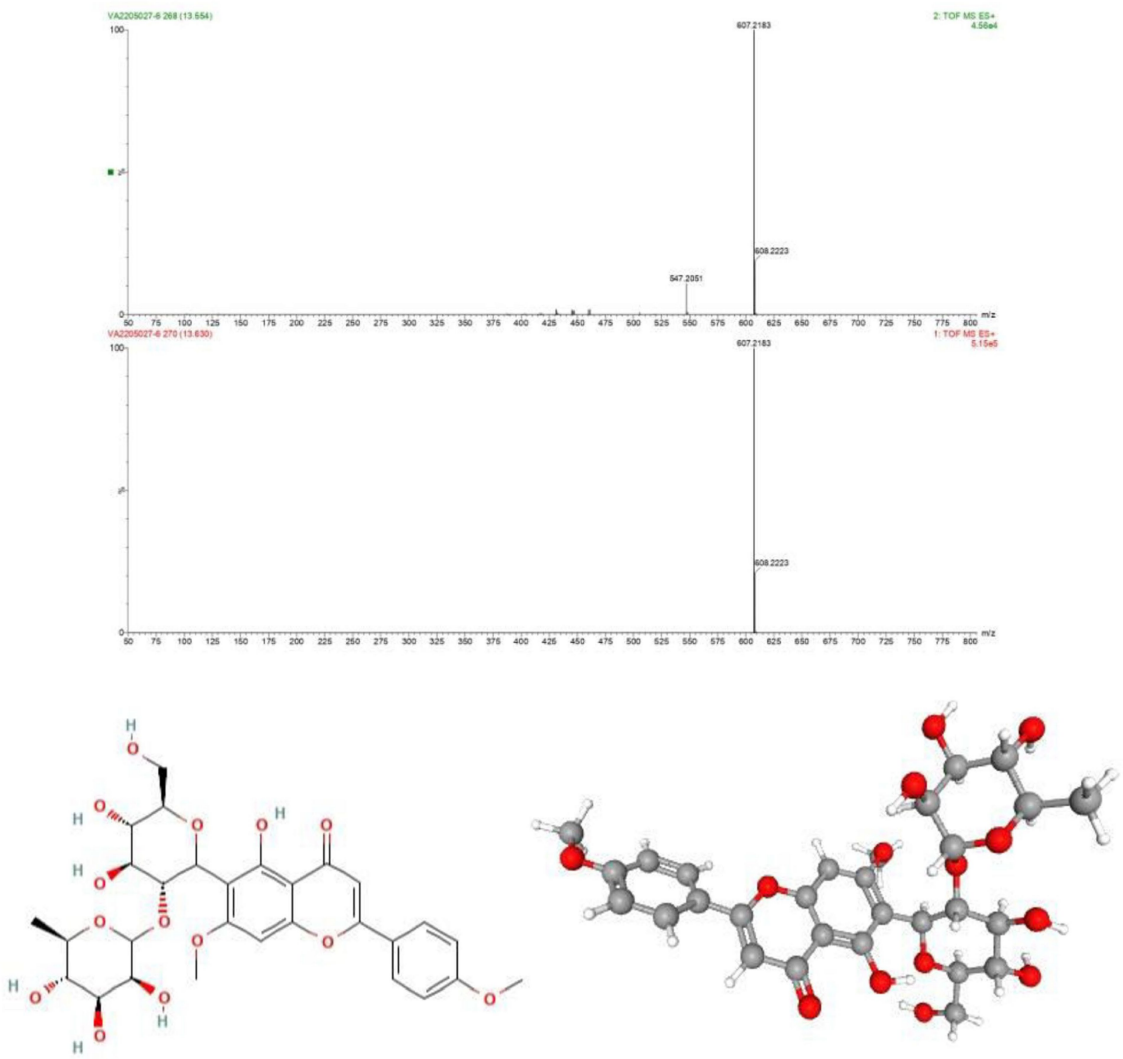
**(A)** Mass confirmation of Embinin, **(B)** 2D structure of Embinin, **(C)** 3D structure of Embinin.

As discussed above the Sephadex G-10 eluted peak protein fraction was further subjected to LCMS analysis. The LCMS analysis and elution profile as shown in Figure 6, revealed the detection of pharmacologically applicable bioactive peptide compounds. With respect to elution peak from LCMS analysis and detection through the NIST, the computer library resulted in the identification of Kinetin-9-riboside and Embinin. The detected Kinetin-9-riboside with 347 Da molecular weight structure and mass confirmation are shown in Figure 7. The mass confirmation and structure of Embinin with a molecular weight of 606 Da showed in Figure 8. These bioactive molecules are well-known for their effective activity in various biological applications.

In a previous study, the therapeutic and biological studies of Kinetin-9-riboside as an immuno-stimulant; immuno-stimulatory activities, and their uses as an adjuvant were reported. Because mutations in induced putative kinase 1 (PINK1) induce severe Parkinson’s disease, there’s a lot of interest in finding small molecules that boost PINK1’s kinase activity. Several studies on the design, synthesis, serum stability and hydrolysis of four kinetin riboside ProTides have been published. These ProTides, in combination with kinetin riboside, activated PINK1 in cells that had not been depolarized by mitochondria. This demonstrates the therapeutic potential of modified nucleosides and their phosphate prodrugs for Parkinson’s disease, the second most common neurodegenerative disease (Osgerby et al., 2017).

Another study found that the epithelial-mesenchymal transition (EMT) is a molecular phenomenon associated with increased vimentin expression and raised activity of transcriptional factors (Snail, Twist) that inhibit E-cadherin. EMT has been linked to prostate cancer metastatic potential, therapy resistance, and poor outcomes. Kinetin riboside (KR) is a naturally occurring cytokinin with effective anticancer activity against several human cancer cell lines. mRNA and protein levels of AR, E-, N-cadherins, Vimentin, Snail, Twist, and MMPs were measured using Western Blot and RT-PCR or RQ-PCR techniques to determine the effect of KR on human prostate cell lines.KR inhibited the growth of human prostate cancer cells and, to a lesser extent, normal cells. The cell type and androgen sensitivity determined this effect. KR also decreased the level of p-Akt, which is involved in androgen signaling modulation. When cancer cell lines are exposed to KR, the anti-apoptotic Bcl-2 protein is down-regulated, whereas the Bax protein is up-regulated. KR was involved in E-cadherin re-expression as well as pivotal changes in cell migration. Taken together, the findings suggest that, for the first time, KR can be anticipated as a factor for signaling pathway regulation that involves the inhibition of the development of aggressive forms of prostate cancer, potentially leading to future therapeutic interventions. As a result, research indicates that KR is an effective inhibitor of EMT in human prostate cells (Thakor et al., 2016; Dulińska-Litewka et al., 2020).

Whereas Embinin is a C-Glycosyl flavone and has a wide therapeutic applications in cardiovascular diseases (Ivkin et al., 2018). Another study reports the production of Embinin frompetals of *Iris germanica* Linnaeus and *Iris lactea* Leaves (Kawase and Yagishita, 1968; Chen et al., 2018). Our study elucidates the cytotoxicity activity of *G. mysorens* bioactive peptide as characterized by LCMS/MS revealed the presence of Kinetin-9-Riboside and Embininin the peptide fraction showing its antiproliferative effect on the prostate cancer cell line. Thus microbial-originated intracellular peptides have potential antimicrobial (AMPs) and anticancer (ACPs) have been significantly substantiated in our studies.

## Conclusion

The present study is illustrative for exploring untapped mangrove habitat in the Mangalore region of Karnataka. In our study, we could demonstrate that mangrove *G. mysorens* is an efficient microbe to produce bioactive compounds and enzymes responsible for both antimicrobial and anticancer activity. The antimicrobial potentiality was detailed in our previous article. In this present study; anticancer activity on prostate cancer cell lines and to treat various other related ailments. Peptides from reliable sources such as *Actinomyces* could be demonstrated as having dual roles as AMPs as well as ACPs. Hence, this study supports and proves that the genus *Glutamicibacter* is an effective microbial group for the isolation of peptides to treat multidrug-resistant pathogens.

## Data availability statement

The datasets presented in this study can be found in online repositories. The names of the repository/repositories and accession number(s) can be found at: NCBI - MW647910.

## Author contributions

YK conducted the research and wrote the manuscript. MI, SK, RD, and CA performed the data analyses and reviewed the manuscript. SK, YK, KR, MAh, MAl, MH, AA, SS, and MM edited and reviewed the manuscript. MI provided experimental support, planned, supervised, and organized the experiment, and wrote and reviewed the manuscript. All authors contributed to the article and approved the submitted version.

## Funding

This work is supported by Science & Engineering Research Board, DST, Govt. of India and Vision Group of Science and Technology Govt. of Karnataka by providing financial and equipment grants.

## Conflict of interest

The authors declare that the research was conducted in the absence of any commercial or financial relationships that could be construed as a potential conflict of interest.

## Publisher’s note

All claims expressed in this article are solely those of the authors and do not necessarily represent those of their affiliated organizations, or those of the publisher, the editors and the reviewers. Any product that may be evaluated in this article, or claim that may be made by its manufacturer, is not guaranteed or endorsed by the publisher.
